# Investigating the effect of a herbal cream containing rose essence in an animal model of eczema

**DOI:** 10.22038/AJP.2024.25132

**Published:** 2025

**Authors:** Ali Reza Malayeri, Anayatollah Salimi, Fariba Iraji, Susan Sabbagh, Neda Shakerian, Mobin Khaledi

**Affiliations:** 1 *Medicinal Plant Research Center, Ahvaz Jundishapur University of Medical Sciences, Ahvaz, Iran*; 2 *Nanotechnology Research Center, Ahvaz Jundishapur University of Medical Sciences, Ahvaz, Iran*; 3 *Department of Dermatology, School of Medicine, Isfahan University of Medical Sciences, Isfahan, Iran*; 4 *Department of* * Anatomical Sciences,* *Faculty of Medicine, Dezful University of Medical Sciences, Dezful, Iran*; 5 *Department of Clinical Biochemistry, Faculty of Medical Sciences, Dezful University of Medical Sciences, Dezful, Iran*; 6 *Department of Experimental Sciences, Dezful Branch, Islamic Azad University, Dezful, Iran*

**Keywords:** Eczema, Rose extract, Dinitrochlorobenzene, Small laboratory mouse, Rosa Spp

## Abstract

**Objective:**

Eczema, a common inflammatory skin condition characterized by itching and dermatitis, can significantly impact the quality of life. While conventional treatments exist, there is interest in exploring natural alternatives. This study investigates the efficacy of a rosehip extract-based cream in mitigating eczema symptoms in a mouse model.

**Materials and Methods:**

Thirty-five mice were divided into five groups: control, dinitrochlorobenzene (DNCB)-induced eczema (model group), and treatment groups receiving placebo, betamethasone cream (positive control), or rosehip extract cream. Following topical treatment for four weeks, the animals were euthanized, and their skin was evaluated for inflammation, moisture, and thickness. Histopathological analysis was performed using hematoxylin and eosin (H&E) staining.

**Results:**

Compared to the control group, DNCB treatment significantly increased inflammation, erythema (redness), dryness, and epidermal thickness. Notably, topical application of the rosehip extract cream significantly reduced these eczema-associated parameters, demonstrating efficacy comparable to the positive control (betamethasone cream).

**Conclusion:**

This study suggests that a topical cream formulated with rosehip extract may be a promising therapeutic strategy for alleviating eczema symptoms. The anti-inflammatory and potentially regenerative properties of rose extract warrant further investigation for the development of natural eczema treatments.

## Introduction

Atopic dermatitis (AD) is a chronic, prevalent skin disease characterized by intense itchiness, unusual excessive skin cell growth, redness, and a rash known as eczema (Wang et al., 2021). Atopic dermatitis (AD) affects approximately 3% of infants, 20% of children, and 3-10% of adults globally (Lipman et al., 2021). The prevalence rate in high-income countries is significantly higher, with up to 20% of children and 10% of adults being impacted by the disorder (Nagavarapu, 2021).

Atopic dermatitis is an intricate and multifaceted ailment that is typified by the presence of impaired skin barrier function and irregular immune system reaction (Patil et al., 2022). Specific immunological factors such as the disruption of the epidermal barrier and abnormal cytokine production, have been identified as causal factors in AD (Moosbrugger-Martinz et al., 2022). Numerous factors, including allergic triggers such as food allergies, contact allergens, irritants, microorganisms, and disrupted epidermal barriers, have been shown to trigger epidermal keratinocytes to initiate an inflammatory response and generate pro-inflammatory mediators including cytokines and chemokines (Simon et al., 2019; Guan et al., 2023). According to available scientific reports, the onset and progression of AD can be traced back to the interplay of various factors including genetic predisposition, exposure to natural allergens, prevailing climatic conditions, and possible exposure to environmental pollutants (Stefanovic et al., 2020). Multiple research efforts investigated the relationship between these factors and AD and evaluated the underlying physiological mechanisms involved (Gu et al., 2023; Singh et al., 2022).

In the context of eczema therapy, topical corticosteroids and topical calcineurin inhibitors represent primary modalities for managing inflammation (Devasenapathy et al., 2022). Conversely, the topical administration of moisturizers serves as a therapeutic approach to address the underlying disorder (Pena et al., 2023). Systemic treatments such as the administration of oral cyclosporine, are employed to support and enhance skin barrier function (Pandey et al., 2022). Numerous factors and exposure to ultraviolet radiation have been identified as potential contributors to the development of severe, treatment-resistant cases of the disease; however, in the majority of instances, a distinct etiology can be discerned (Wang et al., 2022). The identification of pertinent underlying factors and implementation of effective strategies is crucial in combatting the aforementioned issues.

Rosa Spp essential oil is composed of multiple compounds including anthocyanin 5, 3 diglycoside, kaempferol, quercetin, galactoside, arabinoside, and the monoterpene alcohols citronellol and geraniol, as well as the sesquiterpene nonadecane and henicosan (Alizadeh and Fattahi, 2021). Utilization of antioxidants has been suggested as an effective measure to mitigate the risk of cardiovascular disorders (Soto et al., 2020). Furthermore, this botanical species has been employed for medicinal purposes, notably for the management of conditions such as allergic reactions, respiratory afflictions, sleep disorders, and as an anxiolytic agent (Cock and Van Vuuren, 2020). Rose is endowed with several properties that make it efficacious against free radicals and inflammatory cells (Lin et al., 2022). Its anti-cancer characteristics, capacity to prevent gene mutations, and antioxidant activity are also noteworthy attributes (Qi et al., 2022).

Until now, atopic dermatitis has been regarded as an untreatable skin condition, and the usual approach to managing it is through the use of topical steroids, along with systemic immunosuppressive agents and anti-histamines (Mandlik and Mandlik, 2021). The available therapeutic options are imperfect and have limitations due to their association with side effects, rebound phenomenon, or intermittent recurrences (Sun et al., 2023). Numerous unconventional treatments are employed for managing AD, yet these approaches tend to lack substantial scientific backing and can frequently result in more adverse reactions (Wollenberg et al., 2020). Despite this, a recent tendency in the realm of drug development involves utilizing untreated, organic substances to alleviate AD (Singh et al., 2022). It is assumed that the objective behind these endeavors is to intelligently and efficiently create drugs utilizing organic resources. These drugs can serve as standalone treatments or can be used in combination with other therapies to amplify their overall efficacy. To date, scant research has explored the impact of a botanical cream that comprises rosehip extract on the management of eczema. In this study, the objective was to ascertain the impact of a herbal cream comprising rose extract in an animal model of eczema.

## Materials and Methods

The present study conducted animal experimentation following the prescribed protocol of the Institutional Animal Care and Use Committee (IACUC) of the Laboratory Animal Research Center and approved at Ahvaz Jundishapur University, Iran (IR.AJUMS.ABHC.RE.1400.037).  BALB/c mice (6 to 7 weeks-old) were purchased from Orient Bio (Ahvaz Jundishapur University of Medical Sciences, Iran). Each cage housed five mice at a controlled (21–25°C), 12 hr light–dark cycle, and relative humidity (45–65%). Food and water were available ad libitum.

### Mouse population

35 Balb/C mice, aged six to seven weeks, were randomly assigned to five groups namely:

1- Positive control group (mice with eczema),

2- Negative control group (mice with eczema treated with Oserin without rose essence),

3- Control (healthy mice).

4- Herbal cream group (mice with eczema treated with Oserin containing and rose extract).

5- Betamethasone group (mice with eczema treated with betamethasone)

### Extracting essential oil and making creams

This study utilized rose petals obtained from reputable sources. After pulverization, the essential oil was extracted using a steam distillation apparatus. In this process, steam was passed through the rose petals, causing the rose oil to evaporate and separate from the plant material. The condensed vapor was collected as rose essential oil. Finally, this rose essential oil was incorporated into a moisturizing cream base called Oserin to create a uniform mixture. The ingredients of Oserin cream base were; cholesterol, Stearyl Alchohol, White wax and White petrolatum.

### Preparation of sensitization solution

 Initially, a combination of olive oil and acetone in a ratio of 1:3 was utilized to produce two solutions from dinitrochlorobenzene (DNCB) powder, with concentration levels of 0.5% and 1%. For this purpose, to prepare a 0.5% DNCB solution, 250 mg of DNCB granules were weighed with a digital scale and poured into the Falcon tube. The substance was dissolved by employing a mixture of acetone and olive oil with a proportion of 3:1 (consisting of 57.3 ml of acetone and 12.3 ml of olive oil) that was cautiously dispensed onto the substance via a Pasteur pipette. The process of homogenization was carried out thoroughly by utilizing a shaker. The procedure followed for preparing a 1% DNCB.

### Sensitization process

**Figure 1:**
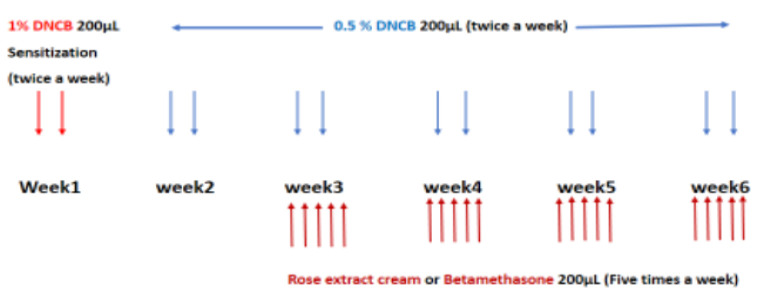
Schematic figure of drug administration steps

The following method was used to sensitize mice to 1%DNCB and induce eczema according to An HJ et al. (An et al., 2018) (Figure 1).

1. Epilation and initial allergen exposure: In the all of experimental groups, the dorsal (back) area of the mice was shaved (epilated) on day one. A swab soaked in a 3:1 mixture of acetone and olive oil was then applied to the shaved area of healthy control group on days 1, 4.

2. Sensitization with DNCB, to induce the development of AD, a solution of approximately 200 μl of 1% DNCB was applied to the dorsal skin of the mice belongs to the groups of positive control, negative control, treatment and betamethasone groups on days 1 and 4.

3. Rest period: The mice were allowed to rest for five days after the first sensitization.

4. Second stage of dermatitis induction; on day 9, for all groups except healthy ones, a solution of approximately 200 μl of 0.5% DNCB was applied to the dorsal skin of the mice to induce dermatitis. Healthy control group received mixture of acetone and olive oil. This method was applied two times a week for five weeks (on days 9, 13, 17, 21, 25, 29, 33, 37, 41, 45).

Additionally, the treatment group received topical application of the herbal cream, the betamethasone group received a commercially available betamethasone cream, while negative control group received placebo (cream base, named Oserin without the active ingredient) five times a week for four weeks.

Endpoint: Finally, the mice were deeply anesthetized with 100 mg/kg ketamine and 10 mg/kg xylazine, and blood samples were taken from their hearts. After centrifugation, sera samples were separated and stored at −70°C.

### Scoring the severity of skin lesions

Throughout the day, from the study start to finish, photos were taken of the mice dorsal area. A trained observer scored the mice on the last day to assess disease severity and tissue damage. The score was defined as the sum of the discrete scores graded as 0 (none), 1 (mild), 2 (moderate), and 3 (severe) for each of four signs: erythema, edema/papulation, excoriation, and scaling/dryness. The total dermatitis score ranged from 0 to 12 (Mudde et al., 1990). Skin moisture in the affected area was also measured based on the TEWL (transepidermal water loss) scale (Alexander et al., 2018) at the beginning and end of the study. Additionally, the macroscopic appearance of the wound area in the photos taken from different groups was compared.

### Measurement of Serum IgE Levels, Iterleukin-4 (IL-4) and Interferon-γ (IFN-γ) Cytokine Levels:

The total serum IgE was quantified by sandwich enzyme-linked immunosorbent assay (ELISA) Quantitation Kit (ZellBio GmbH, Germany) according to the manufacturer’s protocol. The serum concentrations of the cytokines (IL-4 and IFN-γ) were also quantified using a mouse cytokine enzyme immunoassay kit (ZellBio GmbH, Germany). 

### Histopathological study

The samples of skin tissue from the mice back were fixed in 10% formalin, then processed by embedding them in paraffin wax, slicing them into sections of 5 micrometers thick, and staining the sections with  Hematoxylin-Eosin (HE) and giemsa (Salian, 2021). Giemsa staining was done for mast cells. A pathologist then by using a light microscope, measured the thickness of epidermis, counted the number of lymphocytes and mast cells in the skin dermis. The cell counts were performed in twenty consecutive microscopic fields at 400x magnification.

### Statistical analysis

The mean and standard deviation of three determinations were used to display the data values of the results. The analysis of variance (ANOVA) was utilized to make comparisons, with a significance level of less than 0.05 being deemed noteworthy. All experiments were done in triplicate, and the data are plotted as the mean±SD.

## Results

### General findings

**Figure 2:**
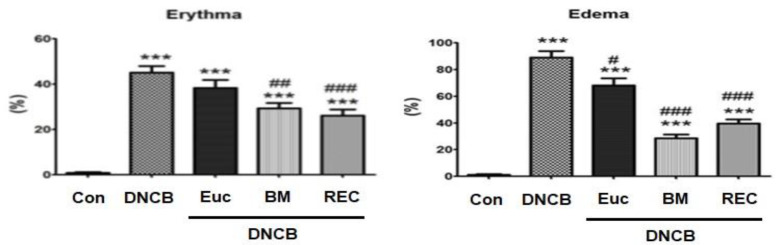
Percentage of edema and erythema of the skin tissue in different groups. *Significant difference with Control group (*p<0.05, **p<0.01, and ***p<0.001). #Significant difference with eczema group) DNCB( (#p<0.05, ##p<0.01, and ###p<0.001). Con: Control, DNCB: Dinitrochlorbenzene, Eue: Eczema+Placebo, BM: Eczema+ BTZ (betamethasone), REC: Eczema+ Ros (Rose extract cream).

In this study, the results showed that the amount of edema and erythema of the skin tissue in the group receiving DNCB increased significantly compared to the control group (p<0.001). Furthermore, the findings indicate that the topical application of rosehip extract cream over a period of four weeks yielded a statistically significant reduction in the levels of edema and erythema observed in the cutaneous tissue when compared to the group that solely received DNCB (p<0.001). The group receiving betamethasone cream also showed a significant decrease in the amount of edema and erythema (p<0.001). Importantly, there was no significant difference between the group receiving betamethasone cream and the group receiving rosehip extract in terms of skin tissue edema and erythema (p<0.001) (Figure 2). 

### Rose cream's effect on dry and thick epidermis in mice

The findings indicated a statistically significant increase in the dryness and thickness of the epidermis of the skin tissue in DNCB-treated mice when compared to the control group (p<0. 001).

**Figure 3:**
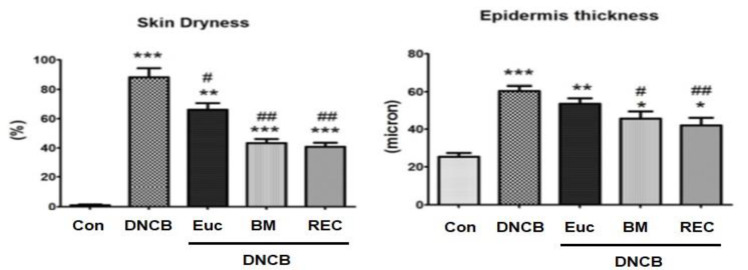
The amount of skin dryness (a) and, epidermal thickness (b) in skin tissue in different groups. *Significant difference with Control group (*p<0.05, **p<0.01, and ***p<0.001). #Significant difference with eczema group) DNCB( (#p<0.05, ##p<0.01, and ###p<0.001).Con: Control, DNCB: Dinitrochlorbenzene, Eue: Eczema+Placebo, BM: Eczema+ BTZ (betamethasone), REC: Eczema+ Ros (Rose extract cream).

In addition, the results show that using a cream containing rose extract for four weeks led to a significant reduction in the dryness and thickness of the skin's epidermis, in contrast to the group that only used DNCB (p<0.001). The group receiving betamethasone cream also showed a significant reduction in the level of skin tissue dryness. The group receiving betamethasone cream also had a significant reduction in the thickness of the epidermis shows (p>0.05). In terms of dryness level and skin epidermis thickness, there was no significant difference between the group receiving betamethasone cream and the group receiving rose extract (p>0.05)(Figure 3(.

### The effect of rose extract cream on the reduction of skin moisture in mice

The study demonstrated that the reduction of skin moisture in the group exclusively treated with DNCB experienced a substantial increase in comparison to the control group (p<0.001).

**Figure 4:**
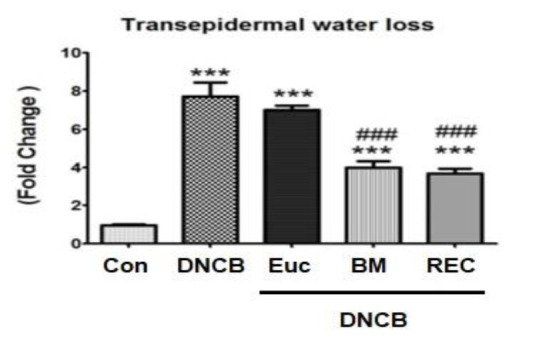
The amount of transepidermal water loss in skin tissue in different groups. *Significant difference with Control group (*p<0.05, **p<0.01, and ***p<0.001). #Significant difference with eczema group) DNCB (#p<0.05, ##p<0.01, and ###p<0.001).Con: Control, DNCB: Dinitrochlorbenzene, Eue: Eczema+Placebo, BM: Eczema+ BTZ (betamethasone), REC: Eczema+ Ros (Rose extract cream).

Also, the results showed that the use of rose extract cream for four weeks increased skin moisture and this index (decrease in skin moisture) compared to the group receiving DNCB alone decreased significantly (p<0.001). Additionally the group receiving betamethasone cream showed a significant decrease of skin dryness as compared to DNCB. In terms of the amount of skin moisture reduction, there was a difference between the group receiving betamethasone cream and the group receiving rose extract, but there was no significance (p>0.05) (Figure 4(.

### Measurement of inflammatory factors

#### The effect of rose extract cream on the number of mast cells and lymphocytes in the skin tissue of small mice laboratory

The results indicated a significant increase in mast cells and lymphocytes in the skin tissue with DNCB compared to the control group) p<0.001). After four weeks of using rose extract cream, there was a marked decrease in the number of mast cells and lymphocytes in the dermis of skin tissue compared to the DNCB-only group. Betamethasone cream decreased mast cells and lymphocytes in skin tissue. In terms of the number of lymphocytes and mast cells in dermis, there was a significant difference between the groups of DNCB and controls, but there wasn’t statistically significant between group receiving betamethasone cream and group receiving rose extract (p>0.05). 

**Figure 5:**
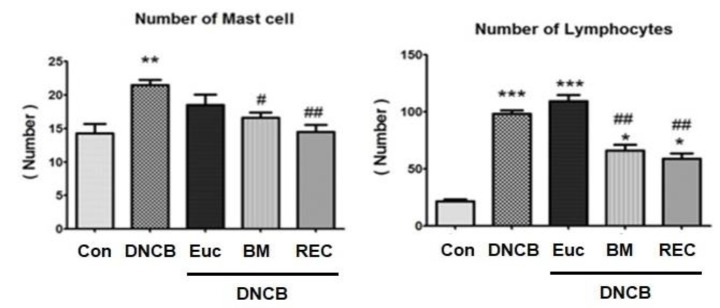
The number of mast cells (a) and, lymphocytes (b) in skin tissue in different groups. *Significant difference with Control group (*p<0.05, **p<0.01, and ***p<0.001). #Significant difference with eczema group) DNCB( (#p<0.05, ##p<0.01, and ###p<0.001).Con: Control, DNCB: Dinitrochlorbenzene, Eue: Eczema+Placebo, BM: Eczema+ BTZ (betamethasone), REC: Eczema+ Ros (Rose extract cream)

**Figure 6:**
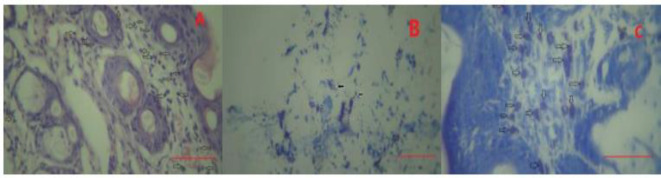
Photomicrograph of inflammatory cells in dermis layer. A: white arrows indicate population of lymphocytes. (H&E staining, ×400). B: black arrows shows degranulation of mast cells in skin dermis layer, (Giemsa staining, ×400). C: white arrows shows aggregation of mast cells in dermis layer (Giemsa staining, ×400), scale bar; 100µm.

Figures 5a and b show the population of mast cells and lymphocytes in different groups. Figure 6 shows the histopathology images of inflammatory cells in the dermis layer of the skin.

### Rose extract cream's effect on immune markers in mice skin

**Figure 7:**
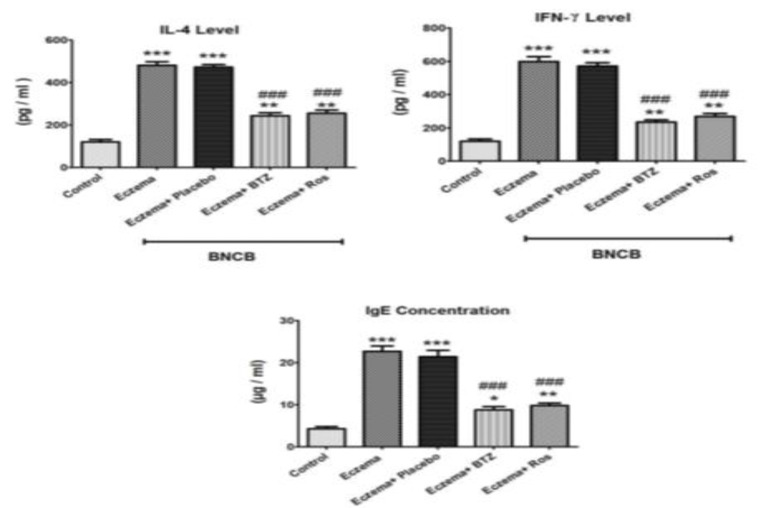
Levels of inflammatory markers in skin tissue of different groups.*Significant difference with Control group (*p<0.05, **p<0.01, and ***p<0.001). #Significant difference with eczema group) DNCB( (#p<0.05, ##p<0.01, and ###p<0.001). Ros: Rose extract cream, BTZ: betamethasone, DNCB: Dinitrochlorbenzene.

This study showed that DNCB led to a significant increase in the levels of interleukin-4, interferon gamma and immunoglobulin E in the skin tissue compared to the control group (p<0.001). After 6 weeks of using the rose extract cream, a significant decrease of certain skin tissue factors was observed compared to the DNCB-only group. Betamethasone cream reduced inflammatory markers in skin tissue. There was a significant difference in interleukin 4, interferon gamma, and immunoglobulin E between the betamethasone cream group and rose extract group (p<0.05) (Figure 7).

### Results of histopathological studies

#### Qualitative results of histopathological studies

**Figure 8:**
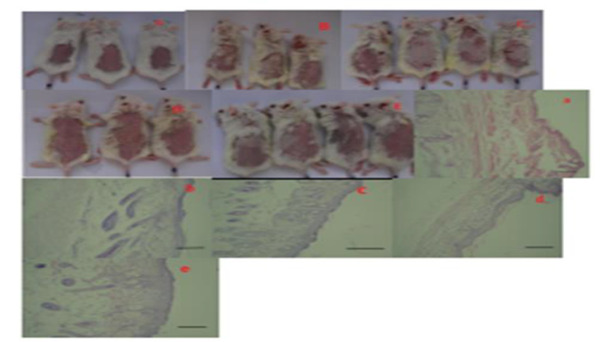
(A) Skin of mice in control group (macroscopic view). (B) Skin of mice in the dinitrochlorobenzene group. (C) Skin of mice in placebo group" (D) Skin of mice in betamethasone group. (a) Skin tissue of mice in control group viewed histopathologically (b) Mouse skin tissue in Dinitrochlorobenzene group. (c) Skin tissue of mice in the placebo group viewed histopathologically stained with H& E. (d) Skin tissue of mice in betamethasone group viewed histopathologically. (e) Skin tissue of mice with rose extract. Scale bar; 100µm.

In the macroscopic examination, skin lesions and eczema in the group receiving DNCB was completely obvious. Dermatological application of rose extract cream and betamethasone cream reduced these lesions and the appearance of the skin was similar to the control group. In the control group, the structure of the tissue was completely normal and no lesions were observed (Figure 8a). Dinitrochlorobenzene group showed severe tissue damage with increased epidermis thickness and inflammatory cell infiltration )Figure 8b .(In the placebo group, the amount of lesions was almost similar to the positive control group. "The thickness of the epidermis increased and the infiltration of a large number of inflammatory cells was observed) Figure 8c (. Betamethasone group had similar lesion amount as negative control. Epidermis thickness and inflammation reduced vs. DNCB group )Figure 8.( Rose extract group had similar lesion amount as negative control group. Epidermis thickness and inflammatory cells infiltration decreased compared to DNCB group (Figure 8e.(

In addition, the photomicrographs of histopathological slides of different groups are shown in Figures 8A-E.

## Discussion

Dinitrochlorobenzene (DNCB) is known to elicit hypersensitivity contact dermatitis (CHS) in various animal models, thereby serving as a valuable tool to examine the underlying mechanisms of this condition (Gilhar et al., 2021). It is hypothesized that DNCB or comparable chemical agents possess the ability to react with various skin proteins and subsequently establish covalent conjugates, thus serving as an immunogenic component (Brites et al., 2020). Hence, in the current investigation, DNCB was employed as an agent to induce cutaneous eczema. The present study investigated the efficacy of a cream derived from rose extract in ameliorating lesions in mice.

The present study employed various measures to evaluate eczema, including but not limited to the assessment of edema, erythema, dryness of the skin, reduction of skin moisture, and thickness of the epidermis.

The outcomes of the current investigation revealed that the administration of DNCB augmented the aforementioned factors in contrast to the group that did not receive any treatment, thereby providing insight into the impact of DNCB drug on the experimental variables under consideration. Based on macroscopic analysis, the manifestation of dermatological abnormalities was readily apparent in mice receiving DNCB.

Multiple studies suggest that atopic dermatitis disease progression can be primarily attributed to immune response dysfunction, which is attenuated by activation of JAK-STAT signaling (Sarapultsev et al., 2023). The activation of the JAK-STAT pathway is triggered by the action of interleukin-4 and interleukin-5, which assume a critical role in the development of skin injuries associated with AD by facilitating the action of proinflammatory cytokines and angiogenic mediators (Hossain et al., 2021). The results of the preceding experimental investigation demonstrated that interleukin-4 induces heterodimerization of its receptor (IL-4R), leading to the acceleration of tyrosine phosphorylation of the IL-4Rα subunit and triggering the activation of Janus kinase 1 (JAK1) (Frempah, 2019). Inflammation cytokines, namely, tumor necrosis factor-a, interleukin—6, interleukin-10, and interleukin-1β, are involved in AD development and progression (Cox, 2022).

In the current investigation, the quantification of mast cells, lymphocytes, interleukin-4, interferon gamma, and immunoglobulin E within the skin tissue was undertaken to assess the extent of inflammation. Exploration into the inflammatory elements present within cutaneous tissue has demonstrated that the introduction of DNCB at a local level induces the aforementioned effects. There has been a notable rise in the quantity of mast cells, lymphocytes, as well as IL-4 and interferon gamma. The observed increase in Immunoglobulin E levels in the experimental group as compared to the control group serves as an indication of the presence of pronounced cutaneous inflammation in the former group. In addition, histopathological analyzes showed significant changes in skin tissue among the experimental and control groups.

The results of this study align with prior research. Han et al. (2017) conducted a research study in Japan to assess the efficacy of Josamycin in treating trinitrochlorobenzene (TNCB)-induced eczema. During the experiment, mice were subjected to weekly topical application of a 0.8% TNCB solution on their ears and backs for three weeks. This exposure resulted in histopathological impairment, as well as an increase in IgE, Th1 and Th2 levels (Han et al., 2017).

The current investigation has demonstrated that the application of cream derived from rose flower extract resulted in a decrease of edema, erythema, and dryness. The group exposed to DNCB and rose extract cream showed a significant reduction in skin dryness, epidermal thickness, and overall skin health.

Such findings suggest a significant impact of skin irritants on skin physiology and may have implications for the development of protective measures against skin damage. Based on the presence of inflammatory factors, this cream has been found to result in a reduction in mast cell and lymphocyte counts, in addition to decreased levels of interleukin-4, gamma interferon, and immunoglobulin E. The observed impacts were further substantiated through macroscopic and microscopic examinations.

The present investigation yielded promising findings indicating that the cream derived from rose flower extract effectively reduced skin lesions and inflammation in DNCB-induced eczema in mice. This finding aligns with the known properties of rose extract, which possesses antioxidant and anti-inflammatory properties. These properties are thought to contribute to the extract's efficacy by neutralizing free radicals (antioxidant effect) and suppressing inflammatory mediators (anti-inflammatory effect), potentially mitigating the damaging effects of DNCB on skin tissue. Importantly, our results suggest that rose extract cream may be a beneficial topical treatment for eczema, although further studies are warranted to explore the long-term effects and mechanisms of action in greater detail. This study highlights the potential of utilizing natural products like rose extract for the management of eczema.
